# Commentary: EEG beta suppression and low gamma modulation are different elements of human upright walking

**DOI:** 10.3389/fnhum.2015.00380

**Published:** 2015-06-26

**Authors:** Carlos Trenado

**Affiliations:** Institute of Clinical Neuroscience, Heinrich Heine University DüsseldorfDüsseldorf, Germany

**Keywords:** gait, phase transition, modulation, index, analysis

Recently Seeber and colleagues proposed a gait-phase modulation (*GPM*) signal to analyze the modulation of EEG oscillatory amplitudes relative to gait cycle. In particular, *GPM* was defined as a modified version of the previously introduced phase-amplitude coupling index (Canolty et al., 2006) under the assumption of a two-period pattern for studying upright walking:

$$GPM\;=\frac{2}{\sqrt{2}\sigma_{A}(f)}\sum_{n\;=\;0}^{N-1}A(n,f)e^{-2\pi i\frac{2n}{N}}.$$

As it is apparent from the mathematical definition and only after application of the absolute value on the obtained complex value for each frequency *f*, *GPM* enables quantification of phase-locking modulated oscillations from cortical regions in relation to periodic cycles in the frequency domain. Consequently, it potentially facilitates the determination of brain networks implicated in periodic body movements as in the case of upright walking by focusing on synchronization values corresponding to frequency bands of interest.

It is worth mentioning that quantification of brain synchronization through phase-locking dates back to the seminal works by Tass et al. (1998) and Lachaux et al. (1999) who introduced a real-valued bounded index (phase-locking value *PLV*) reflecting phase inter-trial variability between brain signals in the time-domain. By observing *PLV* patterns in relation to behavior, these authors were able to emphasize the role of phase synchronization as a putative mechanism for long-range neural integration in cognitive tasks which together with short-range phase synchrony commonly associated to perceptual binding represents an important principle for brain communication. Different studies highlighting the use of phase-locking in brain signals include: neural network connectivity during resting states in humans by magnetoencephalography (Schmidt et al., 2014), assessment of neural correlates of auditory selective attention reflected in electroencephalographic evoked potentials (Trenado et al., 2009), electroencephalographic correlates of listening effort (Strauss et al., 2010), electroencephalographic epilepsy studies (Mormann et al., 2000), studies dealing with brain computer interfaces (Brunner et al., 2006) and studies dealing with interpersonal body and neural synchronization as a marker of implicit social interaction (Yun et al., 2012).

In spite of the usefulness and relevance of the *GPM* signal as previously stated, its definition does not meet the requirements of a phase-locking index as in the case of *PLV*, namely it should provide a real-value as an output and incorporate a scale factor to obtain bounded values in the interval [0, 1] without the need for pre-normalization. To address these issues while providing a more satisfactory definition of gait phase modulation index analogous to*PLV*, the following definition is suggested:

$$GPM_{\sigma }\;=\frac{2}{N\sqrt{2}\sigma_{A}(f)}\left|\sum_{n\;=\;0}^{N-1}A(n,f)e^{-2\pi i\frac{2n}{N}}\right|,$$

where *N* is the total number of samples per gait cycle, *A* denotes the time-frequency magnitude at a certain frequency *f* and sample point *n*, and σ_*A*_(*f*) denotes its standard deviation. In particular, the normalization factor of *GPM*_σ_ aims to capture the proportion of power variation that is coherent with the carrier signal while in the case of *PLV* such a normalization factor, e.g. the number of samples, is merely for scaling.

Focusing on the aim of *GPM*_σ_, an alternative and perhaps more natural way to quantify gait-phase modulation could be given by estimating the correlation between the time-frequency magnitude modulation *A* and the gait cycle, for instance by calculating the magnitude of the Pearson's correlation coefficient between the complex-valued analytic form of the time-frequency magnitude *A* and the complex-valued representation of the gait cycle.

In contrast to *PLV*, *GPM*_σ_ addresses possible phase interactions between a brain signal and a sinusoidal signal representing a periodic motor movement. One of the main advantages of using phase analysis is the fact that it does not require any statistical assumption about the signals while being more robust against noise. An additional capability of this measure is the possibility to evaluate existing phase lags in the motor cycle which are hidden or overlooked by common correlation coefficients and coherence analysis as emphasized by Seeber et al. (2015).

Based on the previous, it is expected that the usefulness of the gait phase modulation index will be made apparent by future studies targeting extraction of neurophysiological correlates of human gait not only in the case of healthy individuals but importantly in patients with motor disorders.

## Conflict of interest statement

The author declares that the research was conducted in the absence of any commercial or financial relationships that could be construed as a potential conflict of interest.

## References

[B1] Brunner C. Scherer R. Graimann B. Supp G. Pfurtscheller G. (2006). Online control of a brain computer interface using phase synchronization. IEEE Trans. Biomed. Eng. 53, 2501–2506. 10.1109/TBME.2006.881775 17153207

[B2] Canolty R. T. Edwards E. Dalal S. S. Soltani M. Nagarajan S. S. Kirsch H. E.. (2006). High gamma power is phase-locked to theta oscillations in human neocortex. Science 313, 1626–1628. 10.1126/science.1128115 16973878 PMC2628289

[B3] Lachaux J.-P. Rodriguez E. Martinerie J. Varela F. J. (1999). Measuring the phase synchrony in brain signals. Hum. Brain Mapp. 8, 194–208. 10.1002/(SICI)1097-0193(1999)8:4<194::AID-HBM4>3.0.CO;2-C 10619414 PMC6873296

[B4] Mormann F. Lehnertz K. David P. E. Elger C. (2000). Mean phase coherence as a measure for phase synchronization and its application to the EEG of epilepsy patients. Phys. D 144, 358–369. 10.1016/S0167-2789(00)00087-724296277

[B5] Schmidt B. T. Ghuman A. S. Huppert T. J. (2014). Whole brain functional connectivity using phase locking measures of resting state magnetoencephalography. Front. Neurosci. 8:141. 10.3389/fnins.2014.00141 25018690 PMC4071638

[B6] Seeber M. Scherer R. Wagner J. Solis-Escalante T. Müller-Putz G. R. (2015). High and low gamma EEG oscillations in central sensorimotor areas are conversely modulated during the human gait cycle. Neuroimage 112, 318–326. 10.1016/j.neuroimage.2015.03.045 25818687

[B7] Strauss D. J. Corona-Strauss F. I. Trenado C. Bernarding C. Reith W. Latzel M.. (2010). Electrophysiological correlates of listening effort: neurodynamical modeling and measurement. Cogn. Neurodyn. 4, 119–131. 10.1007/s11571-010-9111-3 21629585 PMC2866367

[B8] Tass P. Rosenblum M. G. Weule J. Kurths J. Pikovsky A. Volkmann J. (1998). Detection of n:m Phase Locking from Noisy Data: Application to Magnetoencephalography. Phys. Rev. Lett. 81, 3291–3294. 10.1103/PhysRevLett.81.3291

[B9] Trenado C. Haab L. Strauss D. J. (2009). Corticothalamic feedback dynamics for neural correlates of auditory selective attention. IEEE Trans. Neural Syst. Rehabil. Eng. 17, 46–52. 10.1109/TNSRE.2008.2010469 19211323

[B10] Yun K. Watanabe K. Shimojo S. (2012). Interpersonal body and neural synchronization as a marker of implicit social interaction. Sci. Rep. 2:959. 10.1038/srep00959 23233878 PMC3518815

